# Hollow-Fiber Membrane Contactor for Biogas Recovery from Real Anaerobic Membrane Bioreactor Permeate

**DOI:** 10.3390/membranes12020112

**Published:** 2022-01-19

**Authors:** Qazi Sohaib, Carla Kalakech, Christophe Charmette, Jim Cartier, Geoffroy Lesage, Jean-Pierre Mericq

**Affiliations:** Institut Européen des Membranes, IEM, Université de Montpellier, CNRS, ENSCM, 34090 Montpellier, France; carla_kalakech@outlook.com (C.K.); christophe.charmette@umontpellier.fr (C.C.); jim.cartier@umontpellier.fr (J.C.); geoffroy.lesage@umontpellier.fr (G.L.); jean-pierre.mericq@umontpellier.fr (J.-P.M.)

**Keywords:** biogas recovery, methane recovery, AnMBR permeate, membrane degassing, hollow-fiber membrane, gas separation

## Abstract

This study demonstrates the application of hollow-fiber membrane contactors (HFMCs) for the recovery of biogas from the ultrafiltration permeate of an anaerobic membrane bioreactor (AnMBR) and synthetic effluents of pure and mixed CH_4_ and CO_2_. The developed membrane degassing setup was coupled with a pilot-scale AnMBR fed with synthetic domestic effluent working at 25 °C. The membrane degassing unit was able to recover 93% of the total dissolved CH_4_ and 83% of the dissolved CO_2_ in the first two hours of permeate recirculation. The initial recovery rates were very high (0.21 mg CH_4_ L^−1^ min^−1^ and 8.43 mg CO_2_ L^−1^ min^−1^) and the membrane was able to achieve a degassing efficiency of 95.7% for CH_4_ and 76.2% for CO_2_, at a gas to liquid ratio of 1. A higher mass transfer coefficient of CH_4_ was found in all experimental and theoretical evaluations compared to CO_2_. This could also be confirmed from the higher transmembrane mass transport resistance to CO_2_ rather than CH_4_ found in this work. A strong dependency of the selective gas transport on the gas and liquid side hydrodynamics was observed. An increase in the liquid flow rate and gas flow rate favored CH_4_ transport and CO_2_ transport, respectively, over each component. The results confirmed the effectiveness of the collective AnMBR and membrane degassing setup for biogas recovery. Still, additional work is required to improve the membrane contactor’s performance for biogas recovery during long-term operation.

## 1. Introduction

Wastewater, which is currently becoming a significant source for water reuse, is also a potential renewable energy and nutrient resource in the form of biogas and fertilizers [1,2]. The use of wastewater as a renewable energy resource could become more practical through the development and utilization of economical, energy-efficient, and ecofriendly technologies [3]. Aerobic treatment of wastewater has been practiced for a long time; however, this method has various disadvantages including a high energy demand, high maintenance cost, and large amount of sludge production [4]. Alternatively, anaerobic processes have received significant attention as they could overcome the above-mentioned disadvantages of the aerobic processes.

Domestic wastewater has a chemical oxygen demand (COD) lower or equal to 1 g L^−1^, thus categorized as low-strength wastewater, and requires a significant amount of heat for anaerobic treatment since domestic wastewater is discharged in low ambient conditions. However, if low-strength wastewater is treated at low temperatures, the anaerobic process is very attractive by making it cost effective and more energy efficient [5,6,7].

Anaerobic membrane bioreactor (AnMBR) technology has emerged as a potential alternative for low-strength wastewater treatment [8,9,10] by coupling anaerobic bioreactors and membrane separation. This technology requires low energy input, reduces the footprint, and provides an easy scale-up and selective separation between resources and nutrients [11,12]. This method could generate renewable energy in the form of biogas, containing 50–70% CH_4_ and 30–50% CO_2_ [13,14]. However, the discharged anaerobic permeate still contains a huge amount of dissolved methane (dCH_4_) and dissolved carbon dioxide (dCO_2_), which is desorbed into the atmosphere and contributes to greenhouse gas emissions [15]. The dCH_4_ content in the anaerobic effluent ranges from 10–25 mg L^−1^ while the super saturation indices reach as high as 6.9 [16]. Additionally, according to Crone et al., 2016 [17], the average CH_4_ loss in effluent ranges from 11% to 100% depending on the process temperature and other operating conditions. CO_2_ represents a significant contributor to greenhouse gases, with a recently reported tropospheric concertation of ~400 ppm [18]. CH_4_ emissions also affect the concentration of atmospheric greenhouse gas because it is the second largest contributor to global greenhouse gases (~1834 ppb tropospheric concertation), with a 28-fold higher global warming potential than carbon dioxide [15,18]. In addition to environmental concerns, this significant loss of dCH_4_ in effluent represents a significant loss of the produced biogas, which is an economic loss. Several authors have observed that by efficiently recovering the dissolved methane, the AnMBR could be operated without additional energy input. Crone et al., 2016 [17], applied an energy balance on a staged anaerobic fluidized bed-membrane bioreactor (SAF-MBR) system. The total energy requirement of the process (including recovery, upgrading, and compression of dissolved methane; upgrading and compression of headspace methane; and operation of the anaerobic fluidized-bed bioreactor-anaerobic fluidized membrane bioreactor (AFBR-AFMBR) system) was 1.6 × 10^−4^ kWh/L of treated effluent, while the collective dissolved and headspace methane energy value was 1.6 × 10^−4^ kWh/L of treated effluent. This makes the balanced energy requirement equal to zero kWh/L of the treated wastewater.

Several technologies, such as packed columns, aeration towers, freefall jet towers, and diffused aerators, have been implemented to recover dissolved gases from the liquid stream [19]. The above-mentioned degasification methods mostly involve direct contact between the gas and liquid, which creates operational problems, such as flooding, foaming, and emulsion [20]. Some biological methods have also been implemented to recover dissolved methane from anaerobic effluents, namely, aerobic methane oxidation and aerobic methanotrophy [21]. Alternatively, the implementation of membrane degassing (MD) technology using membrane contactors (MCs) has emerged as a promising method to recover methane from aqueous streams [22].

MCs transfer gas molecules across a porous or dense membrane without dispersion of the two phases. Here, the membrane is used as a support for the gas–liquid interface (porous membranes) or as a barrier between the two phases (dense membranes). High degassing efficiencies can be achieved with compact units of MCs as they provide high volumetric mass transfer coefficients [23]. The separation process with MCs is more advantageous over other conventional process as their avoids the problems of gas separation encountered when using conventional methods, such as flooding, foaming channeling, and emulsion formation [24,25,26]. Due to the enhanced gas transport of porous membranes, they are regarded as being suitable for gas/liquid separations and can effectively reduce the footprint and energy consumption [17]. A notable challenge of porous membranes is that they are more prone to wetting. Liquid solvents can penetrate the pores of a membrane, causing an increase in the membrane mass transfer resistance. Membrane wetting can be caused due to the high pressure drop in the membrane module, the transmembrane pressure being higher than the liquid entry pressure, long-term operation, low hydrophobicity of the membrane, low surface tension of the solvents, and large size of the membrane pores [27,28,29]. Increasing the hydrophobicity of the membrane and decreasing the pore size could prevent pore wetting of the membrane, but it also creates other challenges [30,31,32]. Indeed, an increase in the hydrophobicity of the membrane speeds up the process of fouling (organic and bio) [33,34], which can also change the membrane surface properties and thus favor wetting. On the one hand, reducing the porosity might delay the fouling process, but on the other hand, it might reduce the overall performance by reducing the mass transfer flux due to an increased membrane mass transfer resistance [16,35].

In the past, various authors have worked on the recovery of biogas from anaerobic effluents using membrane contactors. Sethunga et al., 2019 [32], investigated dissolved CH_4_ recovery from synthetic and AnMBR effluent using porous PVDF- and PDMS-modified PVDF membrane contactors. The author reported enhanced CH_4_ flux despite an increased layer thickness. The author also demonstrated the importance of preserving the bulk porosity for the recovery of CH_4_ while coating the membrane surface. Sanchis-Perucho et al., 2020 [36], used a commercial PDMS membrane for the recovery of dissolved CH_4_ from AnMBR effluent. However, the reported CH_4_ flux was very low compared to Sethunga et al., 2019 [32]. This lower flux again justifies the importance of using a porous membrane, as suggested by Sethunga et al., 2019 [32]. The findings suggested a high vacuum pressure (0.8 bar) and low liquid flowrates (833 mL min^−1^) for maximum methane recovery. A macroporous PP membrane was used by Jiménez-Robles et al., 2021 [37], for the recovery of CH_4,_ from synthetic effluent (CH_4_ dissolved in water), by combining vacuum and sweep gas desorption. The author reported higher recovery efficiencies in the combination mode, rather than using vacuum and sweep gas separately. The combined sweep gas and vacuum desorption approach was able to achieve a CH_4_ efficiency of nearly 90%. In a very recent work by Jiménez-Robles et al., 2022 [38], a flat sheet membrane contactor was used in the sweep gas mode to analyze the performance of various (dense and porous) commercial membranes. The author used a synthetic CH_4_-water stream and reported an alteration in the hydrophobicity of membranes, during degassing operation. So far, most of the reported work (including the referenced studies) has either been based on a synthetic mixture or has only focused on the recovery of CH_4_. Real effluent contains a large amount of dissolved CO_2_, which cannot be neglected due to the mentioned critical environmental concerns. Although few works have considered the presence of CO_2_ during recovery analysis, there is still a huge gap regarding a detailed analysis of the simultaneous desorption of CH_4_ and CO_2_ and their binary interaction affecting the transmembrane transport of these gases. Therefore, a detailed study of biogas recovery from real AnMBR effluent in real conditions is required.

In this work, we coupled a membrane contactor degassing system with an anaerobic membrane bioreactor bench-scale unit and synthetic effluent preparation unit to study and compare the recovery of biogas from real AnMBR permeate and synthetically prepared mixtures. The desorption of biogas from both synthetic effluents and real AnMBR permeates was thoroughly investigated. AnMBR ultrafiltration permeate was directly used from the AnMBR unit while synthetic effluents were prepared using pure and mixed CH_4_ and CO_2_. This study considered the recovery of both CH_4_ and CO_2_, simultaneously, by investigating their recovery rates, recovery percentage, transmembrane flux, and theoretical and experimental mass transfer coefficients. Simultaneous recovery allowed us to investigate the selective transport of CH_4_ and CO_2_ and to study and compare their mass transfer coefficients and transmembrane mass transport resistance to each gas component.

## 2. Material and Methods

### 2.1. Materials

Analytical-grade pure methane (CH_4_), carbon dioxide (CO_2_), and mixed gas (60% CH_4_ and 40% CO_2_) were purchased from Linde France. Deionized water from the Millipore Elix^®^ 35 Water Purification System was used.

### 2.2. Effluent Preparation and Analysis

**Figure 1:**
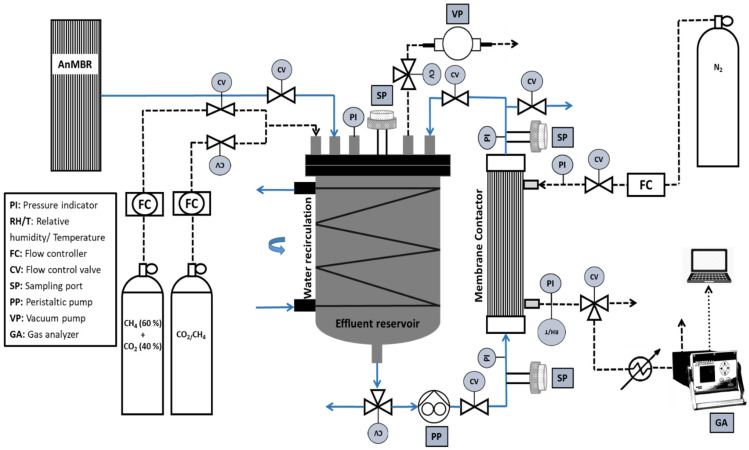
Flow diagram of the developed membrane degassing setup in-line with the anaerobic membrane bioreactor setup (solid lines: liquid stream; dashed lines: gas stream).

Synthetic effluents were prepared directly in an effluent tank using the pure and mixed gas cylinders, as presented in Figure 1. A vacuum pump was used to evacuate the air from the tank. Saturation was achieved by bubbling synthetic gas (for 1 h at 700 mL min^−1^) inside deionized water from the bottom of the tank using a submerged gas diffuser. The gas flow rate was controlled and adjusted using a gas flow meter, the AALBORG GFC17 mass flow controller. The pressure inside the tank during bubbling was monitored by a pressure transmitter STS ATM.1ST/T and was controlled using a control valve. The pressure was normally kept close to the atmospheric level while the temperature of the tank was kept at 25 °C using temperature controlling jacket around the tank.

The real AnMBR permeate (ultrafiltration permeate) was directly transferred to the effluent tank, from the exit of the membrane (AnMBR bench-scale unit), using a peristaltic pump. The main characteristics of the AnMBR permeate are presented in Table 1. The AnMBR unit is briefly described below.

The AnMBR unit was operated at ambient temperature (25 °C) for the treatment of 12 L per day of complex synthetic domestic wastewater (COD/N/P = 400/11/2) [39]. The lab-scale pilot consisted of a 6 L upflow anaerobic membrane bioreactor with a submerged flat sheet Microdyn Nadir^®^ membrane (0.34 m²) with a 0.04 μm pore size and an initial hydraulic resistance of 1.2 10^12^ m^–1^. Additional details regarding the UFmembrane are available in Lahdhiri et al. [40]. A hydraulic retention time of 12h and an organic loading rate of 0.8 kg COD/m^3^/d were applied for more than 6 months before using the permeate for the degassing operation. Total solids and volatile solids were measured in the mixed liquor of the AnMBR tank at about 50 and 15 g/L, respectively.

### 2.3. Degassing

To recover the biogas from the effluents, a membrane contactor-based pilot setup was developed as presented in Figure 1 using porous hydrophobic membranes.

A hollow-fiber membrane contactor module (3M™ Liqui-Cel™ MM-1.7 × 5.5) with a parallel configuration and 0.58 m^2^ effective inner membrane area was supplied by 3M™, USA. The module contains hydrophobic polypropylene hollow fibers with a 40% porosity, potted with polyurethane. The specifications and operating conditions are presented in Table 2 (provided by the manufacturer).

The effluent was recirculated from the effluent reservoir to the lumen side of the module using an industrial peristaltic pump LONGER, G600-1J-1. The liquid flow rate varied from 100–400 mL min^−1^. The lumen side pressure drop was monitored by a differential pressure transmitter, MICROSENSOR, MDM490, which varied from 6.8–28.5 mbar depending on the flow rates. A sweep gas (N_2_) was allowed to flow counter currently through the shell side of the module. The flow rate of the sweep gas varied from 50–200 mL min^−1^ using the gas flow meter, AALBORG GFC17 mass flow controller. The gas side pressure at the inlet and outlet was monitored by a pressure transmitter, STS ATM.ECO. A hygro transmitter, Delta OHM HD48T was also installed at the gas outlet to measure the relative humidity (RH) and temperature of the exit gas. The temperature of the effluent tank was kept constant at 25 °C (similar to the one at which the AnMBR plant was operated) using a temperature-controlling jacket. The effluent was recirculated in a closed loop to the tank until the amount of biogas was degassed. The progress of the desorption was monitored on both the gas and liquid sides, as explained in Section 2.4. After each desorption with AnMBR permeate, the membrane was cleaned with deionized water by the countercurrent flow. Experiments were replicated three times and the data is presented as an average.

### 2.4. Determination of the Desorbed and Dissolved Biogas

The gas side concentration of the biogas at the module gas outlet was measured using a continuous biogas analyzer, Emerson X-Stream Enhanced XEGK. A condenser was installed before the analyzer to avoid water vapors. The analyzer indicated the biogas concentration in the dry gas. The biogas concentration in the wet gas (actual concentration of biogas at the immediate gas side exit) was calculated taking into consideration the water molar fraction and using *RH* (%) at the exit of the module (Equation (1)):

(1) $$y_{H_{2}O}=\frac{\%RH*p_{H_{2}O}^{*}}{100*P}$$

where *y_H2O_* (-) represents the molar fraction of water vapors at the membrane contactor gas side outlet, p*_H__2*O*_ (bar) is the saturated state water vapor pressure, and *P* (bar) represents the total gas pressure at the membrane contactor gas side outlet.

The water vapor molar fraction ranged from 0.026 to 0.03 for all experiments. All of the gas side results presented in this work are based on the wet biogas concentrations.

The liquid side concentrations of the biogas at the module liquid side inlet and outlet were measured by the headspace method as described by other authors [7,42,43]. The gas tight vials used an an 11.6 mL total volume (with a magnetic stirrer inside). Each vial was prepared for 10 min by passing helium gas through it to evacuate the air and finally to retrain helium at ~100 mbar. Helium gas was used as it is the carrier gas present in the gas chromatograph used for analysis. Samples of 6 mL were collected from the sampling port using the gastight syringe from Hamilton, gastight 1010, and injected through the prepared vials. The vials were then stirred at 500 rpm and 25 °C until equilibrium was reached (~10 min). Then, 300 μL of head space gas were collected using a 1 mL Hamilton gastight 1001 syringe, and injected through a PerkinElmer Gas Chromatograph, Clarus^®^ 680, coupled with a thermal conductivity detector (TCD). The TCD temperature was 150 °C. The column used was RESTEK, ShinCarbon ST 100/120 with a 2 m length and 1 mm inner dial. The column temperature varied from 40–200 °C, with an initial pressure of 50 psi. The injection port temperature was kept at 200 °C. The carrier gas (He) flow rate ranged from 10–12 mL min^−1^. The gas chromatograph indicated the biogas concentration in the head space *C_gh_* (mg L^−1^)_._ The dissolved biogas concentration in the liquid phase *C_l_* (mg L^−1^) was calculated from Equation (2):

(2) Cl=Cgh(Vgh+VlH)Vl

where *V_gh_* and *V_l_* represent the head space volume and the liquid volume in the vial, respectively. H (C^*^_gh_/ C^*^_l_) in the equation represents the dimensionless Henry’s law constant for equilibrium between the mass gas (C^*^_gh_) and liquid (C^*^_l_) concentrations inside the vial. The temperature-dependent dimensionless Henry’s law constant *H* (—) was calculated from Equations (3) and (4) [44]:

(3) $$H^{*}\left({\mathrm{mol}\;L}^{-1}{\;\mathrm{bar}}^{-1}\right)=H^{\theta }*exp\left(\frac{-{∆}_{sol}H}{R}\left(\frac{1}{T}-\frac{1}{T^{\theta }}\right)\right)$$

(4) $$H\left(-\right)=\frac{1}{H^{*}R\;T}$$

where *H*^θ^ (mol L^−1^ bar^−1^) and *T*^θ^ (°C) are the reference constants, R (L bar °C^−1^ mol^−1^) represents the gas constant, and ∆*_sol_ H* (j mol^−1^) is the enthalpy of dissolution.

### 2.5. Performance Evaluation and Mass Transfer Calculations

The theoretical concentrations *C_i_* (mg L^−1^) of the dissolved mixed and pure gases in the effluent were predicted based on the gas partial pressure approach using Henry’s law constant H^*^ from Equation (3):

(5) $$C_{i}=H^{*}\;y_{i}\;P_{t}$$

The degassing performance of the MC desorption setup was evaluated based on the membrane recovery percentage, recovery rate (mg L^−1^ min^−1^), degassing efficiency, gas side transmembrane flux, N (mg m^−2^ min^−1^), and experimental mass transfer coefficient (m s^−1^). The membrane biogas recovery (%) and recovery rate (mg L^−1^ min^−1^) over a time span, *t*, were calculated from the dissolved biogas concentration (initial = *C_l_*_-*i*_, after time *t* = *C_l_*_-*t*_) in the effluent tank following Equations (6) and (7):

(6) $$\%\;Recovery_{t}=\frac{C_{l-i}-C_{l-t}}{C_{l-i}}*100$$

(7) $$Recovery\;rate_{t}=\frac{C_{l-i}-C_{l-t}}{t}$$

The membrane degassing efficiency was calculated using the dissolved biogas concentration at the module liquid side inlet *C_l_*_-*in*_ (mg L^−1^) and outlet *C_l_*_-*out*_ (mg L^−1^) using Equation (8):

(8) $$Degassing\;efficiency=\frac{C_{l-in}-C_{l-out}}{C_{l-in}}*100$$

Transmembrane biogas molar flux (mg m^−2^ min^−1^) was calculated using Equation (9).

(9) $$N=\frac{Q_{g-out}C_{g-out}-Q_{g-in}C_{g-in}}{Mg\;A_{o}}=\frac{Q_{l-in}C_{l-in}-Q_{g-out}C_{g-out}}{Mg\;A_{i}}$$

where *M_g_* (mg mol^−1^) represents the molar mass of biogas, *A_o_* (m^2^) is the effective area of the membrane, *C_g_*_-*in*_ (mg L^−1^) is the gas concentration at the module gas side inlet, *C_g_*_-*out*_ (mg L^−1^) is the gas concentration at the module gas side outlet, *Q_g_*_-*in*_ (mL min^−1^) is the gas flowrate at the module gas side inlet, and *Q_g_*_-*out*_ (mL min^−1^) is the gas flow rate at the module gas side outlet.

The overall experimental mass transfer coefficient, *K_exp_* (m s^−1^), was calculated using the following equation [28]:

(10) $$K_{exp}=\frac{Q_{l}\left(C_{l-in}-C_{l-out}\right)}{A_{i}∆C_{lm}}$$

where ∆*C_lm_* is the logarithmic mean of the driving force, which was calculated using Equation (11) [45]:

(11) $$∆C_{lm}=\frac{\left(C_{l-in}-C_{l-in}^{*}\right)-\left(C_{l-out}-C_{l-out}^{*}\right)}{\ln\left(\frac{C_{l-in}-C_{l-in}^{*}}{C_{l-out}-C_{l-out}^{*}}\right)}$$

where C^*^_l__-*in*_ (C^*^_l__-*in*_ = *C_g_*_-*out*_/*H*) and C^*^_l__-*out*_ (C^*^_l__-*out*_ = *C_g_*_-*in*_/*H*) are the liquid phase biogas concentrations in equilibrium with the gas phase outlet (*C_g_*_-*out*_) and inlet (*C_g_*_-*in*_ = 0) concentrations, respectively. The predicted values of H at 25 °C were 28.81 for CH_4_ and 1.22 for CO_2_.

The theoretical overall mass transfer coefficient *K_ov_* (m s^−1^) based on the liquid phase was calculated from the resistance in the series model for the liquid in the lumen side configuration, as presented in Equation (12) [46]:

(12) $$\frac{1}{K_{ov}}=\frac{d_{i}}{Hd_{o}k_{g}}+\frac{d_{i}}{Hd_{lm}k_{m}}+\frac{1}{k_{l}}$$

where *d_i_* (m), *d_o_* (m), and *d_lm_* (m) are the inner tube, outer tube, and log mean diameters; and *k_g_* (m s^−1^), *k_m_* (m s^−1^), and *k_l_* (m s^−1^) represent the mass transfer coefficients of the gas membrane and liquid phases, respectively. H represents the dimensionless Henry’s law constant calculated from Equation (4). For a cylindrical geometry, such as MC, the overall resistance, *R_ov_* (s m^−3^), can be found by adding the resistance in all phases as shown in Equation (13) [37]:

(13) $$R_{ov}=\frac{1}{K_{ov}A_{i}}=\frac{1}{Hk_{g}A_{o}}+\frac{1}{Hk_{m}A_{lm}}+\frac{1}{k_{l}A_{i}}\;$$

where *A_i_* (m^2^), *A_o_* (m^2^), and *A_lm_* (m^2^) are the inner, outer, and log mean membrane mass transfer areas, respectively.

In a similar way, the experimental resistance, *R_exp_* (s m^−3^), was calculated using Equation (14):

(14) $$R_{exp}=\frac{1}{K_{exp}A_{i}}$$

For the gas phase mass transfer coefficient *k_g_*, the correlation from Equation (15) was used [47]:

(15) $$S_{hg}=\frac{k_{g}d_{h}}{D_{g}}=5.85\;\left(1-\phi \right)\left(\frac{d_{h}}{L}\right)R_{e}^{0.6}S_{c}^{0.33}\;0<R_{e}<500;0.04<\phi <0.4$$

For the liquid phase (flowing through the lumen), the mass transfer coefficient *k_l_* was estimated using the Leveque equation [48]:

(16) $$S_{hl}=\frac{k_{l}d_{i}}{D_{l}}=1.62\;{\left(\frac{d_{i}}{L}R_{e}S_{c}\right)}^{1/3}$$

For the gas-filled membrane, the mass transfer coefficient, *k_m_*_,*g*_ was estimated using Equation (17):

(17) $$k_{m,g}=\frac{\varepsilon }{\tau \delta }D_{m,g}$$

The physical parameters used to predict the above-mentioned mass transfer coefficients are listed in Table A1 of the Appendix A.

To predict the effect of pseudo-wetting, the back substitution method was implemented for the pseudo-wetted membrane mass transfer coefficient, *k_m_*_,*w*_, using the experimental mass transfer coefficient *K_exp_* in Equation (10) instead of the overall mass transfer coefficient *K_ov_*:

(18) $$\frac{1}{k_{m,w}}=\left(\frac{1}{K_{exp}}-\frac{d_{i}}{Hd_{o}k_{g}}-\frac{1}{k_{l}}\right)\frac{Hd_{lm}}{d_{i}}$$

The effective gas diffusion coefficient, *D_m_*_,*eff*_, of the pseudo-wetted membrane was calculated from:

(19) $$D_{m,eff}=\frac{k_{m,w}\tau \delta }{\varepsilon }$$

## 3. Results and Discussion

### 3.1. Degassing System Performance

**Figure 2:**
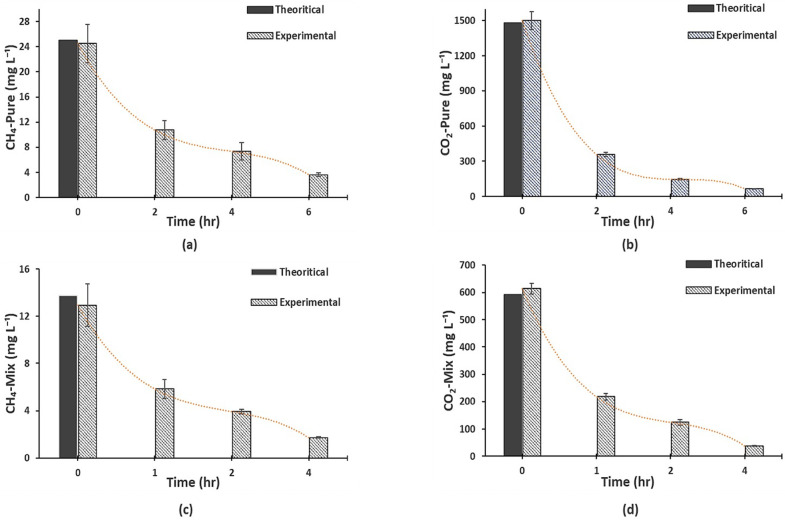
Reduction of the dissolved biogas concentration by membrane degassing in the effluent tank against operational time for (**a**) pure CH_4_, (**b**) pure CO_2_, (**c**) mix CH_4_ (60%), (**d**) mix CO_2_ (40%); Q_l_ = 100 mL min^−1^, Re_l_ = 1.30, Q_g_ = 100 mL min^−1^, Re_g_ = 0.07, T = 25 °C, P_h_ (effluent head space pressure) = 1 bar.

Experiments were first conducted using synthetic effluents with pure CH_4_, pure CO_2_, and a mixture of CH_4_ and CO_2_. The initial biogas concentrations of the prepared synthetic mixtures (effluents) were measured using the head space method explained in the materials and methods section. The theoretical biogas concentrations were calculated based on the gas partial pressure approach and Henry’s solubility law (details are provided in Section 2.4 and Section 2.5) and are presented in Figure 2. Figure 2 shows that the theoretical values are close to the ones measured for the effluents in the effluent tank. The percent difference ranged from 1.3–5%. This validates the methods and protocols followed during the preparation of synthetic mixtures and the equilibrium protocols implemented for the vial preparation for the head space method.

The MC degassing progress was monitored by measuring the dissolved biogas concentration in the effluent tank at various operational times. Figure 2 shows a significant drop in the concentration of dissolved biogas in the effluent with the operational time of degassing. For example, in the first two hours, the concentration of pure dissolved CH_4_ dropped from 24.5 mg L^−1^ to 10.7 mg L^−1^ while the concentration of pure dissolved CO_2_ dropped from 1500 mg L^−1^ to 358.3 mg L^−1^. It is also evident that the concentration drop was faster at the beginning and slowed down with time. The high initial drop could have been caused by the high initial concentration of the dissolved gas in the liquid and hence the high driving force through the membrane. For the same reason, the drop was higher for pure CO_2_ than for pure CH_4_. Similar trends were observed for both CH_4_ and CO_2_ when they were mixed (Figure 2c,d). This significant drop in the concentration of dissolved biogas in the effluent provides evidence that the degassing method (followed here) was effective.

**Figure 3:**
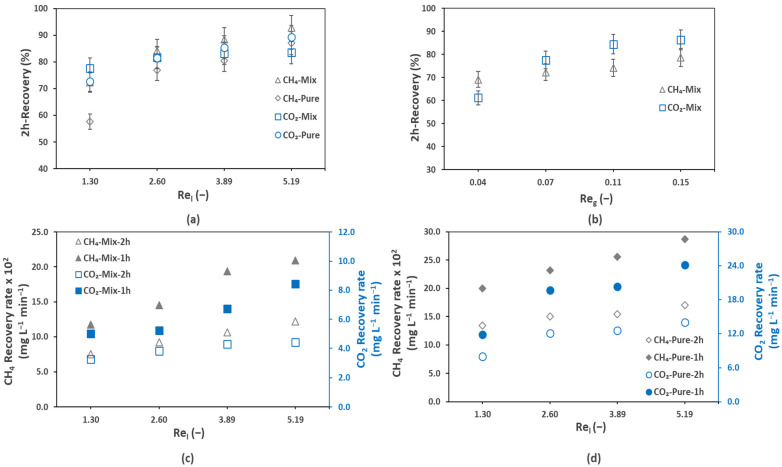
Percent 2 h recovery percentage and average (over a 1 h and 2 h time span) recovery rates of biogas after degassing for pure and mix effluents: (**a**) 2 h recovery percentage at different liquid side Reynold numbers (Re_l_) for a fixed gas side Reynolds number (Re_g_) of 0.07, (**b**) 2 h recovery percentage at different Re_g_ for a fixed Re_l_ of 1.30, (**c**) mix gas effluent recovery rates at different Re_l_ numbers for a fixed Re_g_ of 0.07, (**d**) pure gas effluent recovery rates at different Re_l_ numbers for Re_g_ of 0.07; T = 25 °C.

The recovery percentage (Equation (5)) of the biogas in the first two hours (2h-Recovery percentage) of the degassing operation is presented in Figure 3 for different values of the liquid side Reynolds number, Re_l_ (1.30–5.19), and gas side Reynolds number, Re_g_ (0.07) (Figure 3a), and for different values of Re_g_ (0.04–0.15) and a fixed Re_l_ (1.30) (Figure 3b). By fixing the liquid side Re_l_ at 5.19 and Re_g_ at 0.07, the achieved 2h-recovery percentages were 92.8%, 87.0%, 83.4%, and 89.2% for mix CH_4_, pure CH_4_, mix CO_2_, and pure CO_2_, respectively. The above-mentioned recovery percentages confirmed the recovery of most of the dissolved biogas in the first two hours of the degassing. A significant effect of the Reynold number on the 2h-recovery percentage was also observed. For example, for pure CH_4_ an, increase in the liquid side Reynold number from 1.29 to 5.19 increased the 2h-recovery percentage by 29.4%. The gas side Reynold number had similar effects on the 2h-recovery percentage. An increase of 9.6% and 25.2% in the 2h-recovery percentages was observed for mix CH_4_ and mix CO_2_, respectively, by increasing the gas Reynold number from 0.037 to 0.15.

The CH_4_ and CO_2_ recovery rates (mg L^−1^ min^−1^) were calculated from Equation (7). As the degassing process was not completely in steady state and the degassing rate changed with time, the recovery rate was also not constant. Therefore, we calculated average biogas recovery rate over 1-h and 2-h span of time. The average recovery rate for the mix (Figure 3c) and pure effluents (Figure 3d) is presented in Figure 3 for different Re_l_ (1.30–5.19) and for a fixed Re_g_ (0.07). The recovery rates of the dissolved CO_2_ were very high compared to the dissolved CH_4_, besides the fact that CH_4_ can be easily recovered (degassed) from water. By fixing Re_l_ at 5.19 and Re_g_, at 0.07, the 1h-recovery rates of CH_4_ and CO_2_ were 0.21 and 8.4 mg L^−1^ min^−1^, respectively. The high CO_2_ recovery rates can be explained by the large quantity of CO_2_ dissolved in the effluent due to its nearly 24 times higher water solubility (considering Henry’s solubility law; Equations (3) and (4)) than CH_4_. The 1h recovery analysis gave nearly two times higher values that that of the 2h recovery, which explains why the recovery was faster in the initial hours of degassing. For example, at Re_l_ of 5.19, the mix CH_4_ 2h recovery rate was 0.11 mg L^−1^ min^−1^, nearly half of the 1h recovery rate (0.21 mg L^−1^ min^−1^). Additionally, a significant effect of the Reynolds number on both the 1h and 2h recovery rates was observed. For example, the 1h recovery rate of the mix CH_4_ increased from 0.12 mg L^−1^ min^−1^ to 0.21 mg L^−1^ min^−1^ by increasing Re_l_ from 1.29 to 5.19. The effects of the gas and liquid side hydrodynamics on the membrane degassing efficiency will be further discussed in the following sections.

### 3.2. Membrane Degassing System Efficiency

**Figure 4:**
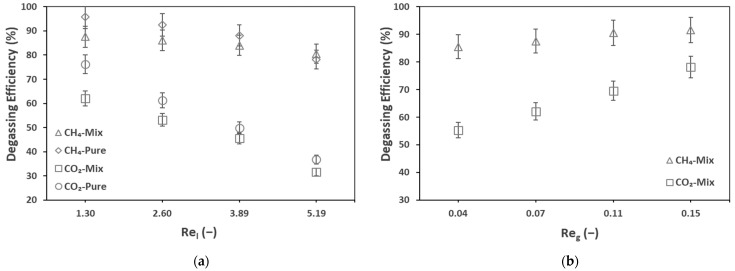
Membrane degassing efficiency for pure and mix effluent, against (**a**) liquid Reynold, Re_l_ = 1.30–5.19, liquid flow rate, Q_l_ = 100–400 mL min^−1^ (**b**) gas Reynold, Re_g_ = 0.07–0.15, gas flow rate, Q_g_ = 50–200 mL min^−1^; T = 25 °C.

In the previous section, the membrane contactor degassing system global performances are discussed. The liquid and gas flow rates (or velocities/Reynolds numbers) are the two most important operational conditions in the operation of membrane contactors. This section presents the effect of the above-mentioned parameters on the degassing efficiency (Equation (7)) of the membrane (between the inlet and outlet of the membrane contactor) for biogas recovery. Figure 4 presents the effect of the liquid flow rate (a) and gas flow rate (b) on the degassing efficiency of the membrane.

Increasing the liquid flowrate decreased the membrane degassing efficiency while the global recovery was increased (see the previous section). For example, at a liquid flow rate of 100 mL min^−1^ (Re_l_ = 1.30), the inlet and outlet dissolved CH_4_ concentrations were 27.7 and 1.2 mg L^−1^, respectively, resulting in a membrane degassing efficiency of 95.8%. This efficiency dropped to 78.1% (5.7 mg L^−1^ dissolved CH_4_ at the membrane outlet) by increasing the liquid flowrate from 100 mL min^−1^ (Re_l_ = 1.30) to 400 mL min^−1^ (Re_l_ = 5.19). This seems to be related to the short residence time of the effluent inside the membrane module at higher liquid flowrates. Similar effects were observed by Sanchis-Perucho et al., 2020 [36], Jiménez-Robles et al., 2021 [37], and Henares et al., 2018 [43], as presented in Table 3. The porous membranes presented in this table had the same pore size of 0.04 µm. Despite causing a decrease in the degassing efficiency, an increase in the liquid flow rate increased the recovery percentage and recovery rate (Figure 3), which confirms that even if the degassing membrane efficiency decreases, the overall performance is enhanced, with an increase in the liquid flow rate. Two phenomena affected the degassing performance: the transmembrane flux (related to the concentration difference/driving force and the mass transfer coefficient) and the liquid retention time. An increase in the liquid flow rate increased the mass transfer coefficient and thus decreased the liquid-side mass transfer resistance. The above-mentioned results justify the dominance of the transmembrane flux over the retention time.

The effect of the gas flow rate was investigated by Cookney et al., 2016 [49], who reported that changing the sweep gas velocity from 0.0015 m s^−1^ to 0.009 m s^−1^ had almost no influence on the degassing efficiency. However, in this work, we observed an influence of the gas flow rate. An increase in the sweep gas flow rate increased the degassing efficiency. Particularly, for CO_2_, increasing the gas flowrate from 50 (Re_g_ = 0.04) ml min^−1^ to 200 mL min^−1^ (Re_g_ = 0.15) increased the degassing efficiency from 55.3% (249.1 mg L^−1^ dissolved CO_2_ at the membrane outlet) to 78.1% (112.6 mg L^−1^ dissolved CO_2_ at the membrane outlet). This might have occurred due to an increase in the transmembrane driving force because of a lower gas side biogas concentration due to the dilution effect at higher sweep gas flow rates.

### 3.3. Analysis of Biogas Flux

**Figure 5:**
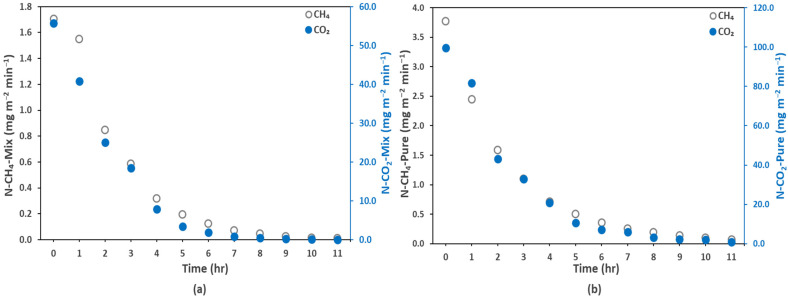
Biogas transmembrane flux for (**a**) mixed gas and (**b**) pure gas effluents against operational time; Q_l_ = 100 mL min^−1^ (Re_l_ = 1.30), Q_g_ = 100 mL min^−1^ (Re_g_ = 0.07); T = 25 °C.

**Figure 6:**
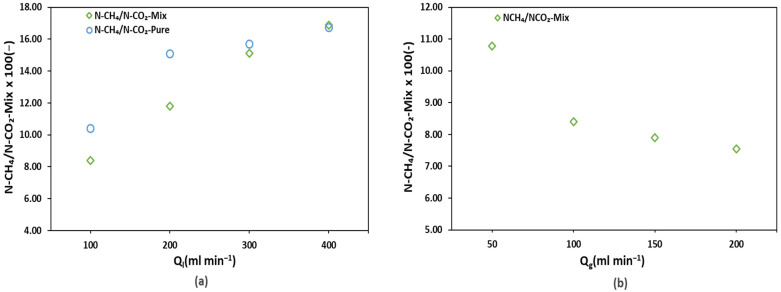
Selective flux transport of CH_4_ over CO_2_ in mix gas and pure gas effluents against (**a**) the liquid flow rates (**b**) gas flow rates.

The transmembrane permeate flux was calculated based on the gas side concentrations (Equation (9)) of the membrane contactor degassing operations measured and presented in Figure 5 and Table 4. The gas stream leaving the module exit consisted of biogas (CH_4_ + CO_2_), sweep gas (N_2_), and water vapor. The presence of water vapor only slightly impacted the biogas flux. The biogas flux values presented in this section represent the actual flux values, which is the wet flux (considering water flux). The percent difference between the wet flux (immediate exit of the membrane contactor) and the dry flux (at the gas analyzer inlet) ranged from 2.6–3.1% based on the percentage of relative humidity (RH). Figure 6 presents the variations of the transmembrane flux for the mix (a) and pure (b) gas effluents with the degassing operation time. Initially, the flux was high for both CH_4_ and CO_2_, which dropped to a lower value in the first 2–3 h of the membrane degassing operation. This confirms that initially, the flux was very high and the recovery was very fast, which dropped with the operation time and became slower during the later hours. Initially, the mix CH_4_ flux was 1.7 mg m^−2^ min^−1^ while for pure CH_4_, it was 3.8 mg m^−2^ min^−1^. For mix CO_2_, an initial flux of 55.7 mg m^−2^ min^−1^ was observed while for pure CO_2_, it was 99.6 mg m^−2^ min^−1^. This is again related to the decrease of the transmembrane driving force versus time.

Table 4 presents the effect of the gas and liquid flow rates on the CH_4_ transmembrane flux. It can be observed that at higher liquid flow rates, the flux was high, which again confirms that even if the membrane degassing efficiency decreases (Figure 4), the overall performance is enhanced with an increase in the liquid flow rate. For example, for pure CH_4_, the reported flux at 100 mL min^−1^ (Re_l_ = 1.30) was 3.8 mg m^−2^ min^−1^, which increased to 11.1 mg m^−2^ min^−1^ at 400 mL min^−1^ (Re_l_ = 5.19). Similar effects were confirmed by Cookney et al., 2016 [49] and Wongchitphimon et al., 2017 [50]. The gas flow rate was also observed to influence the biogas flux. For mix CH_4_, the reported flux at 50 mL min^−1^ (Re_g_ = 0.04) was 1.5 mg m^−2^ min^−1^, which increased to 1.9 mg m^−2^ min^−1^ at 200 mL min^−1^ (Re_g_ = 0.15).

Table 5 presents a comparison of the CH_4_ flux between the current study and studies from the literature. This work recorded a CH_4_ flux of 11.1 mg m^−2^ min^−1^ at a liquid flow rate of 400 mL min^−1^ (Re_l_ = 5.19) and gas flow rate of 100 mL min^−1^ (Re_g_ = 0.07). Sanchis-Perucho et al., 2020 [36] comparatively reported a very low flux of 2.4 mg m^−2^ min^−1^ while maintaining high liquid flowrate of 833 mL min^−1^. The low flux seems to be related to the high mass transfer resistance when using a PDMS dense membrane instead of a porous membrane. Sethunga et al., 2019 [32] reported a flux of 9.63 mg m^−2^ min^−1^, applying a very high liquid velocity compared to our work. However, this author reported a high flux value (18.3 mg m^−2^ min^−1^) using a modified PVDF membrane in similar operating conditions.

To observe the selective transport of CH_4_ over CO_2_ and to analyze whether the transport of both gases could be affected in the case of mix gas effluents (in comparison with pure gas effluents), the ratios of CH_4_ flux to CO_2_ flux were plotted (Figure 6) against various liquid (a) and gas (b) flowrates. The figures below explain whether the presence of CH_4_ and CO_2_ as soluble gases in the effluent influences their simultaneous transport in the membrane desorption setup. It is evident from Figure 6a that the CH_4_ to CO_2_ flux ratios for pure and mix gas effluents are not similar, thus selective transport is affected by the presence of other gas. the CH_4_ to CO_2_ flux ratio is higher for pure gas than for mix gas.

Mix gas transport favored the transport of CO_2_ over CH_4_ compared to pure gas transport. On the one hand, we can observe that an increase in the liquid flow rate favored CH_4_ flux over CO_2_, as increasing the liquid flow rate resulted in an increase in the CH_4_ to CO_2_ flux ratio. On the contrary, an increase in the gas flow rate decreased the CH_4_ to CO_2_ flux ratio, thus favoring CO_2_ flux. An increase in both the liquid and gas flow rates decreased the transmembrane resistance (on liquid and gas side). The reason for the liquid flow rate favoring CH_4_ transport might be because CH_4_ can be easily degassed from water compared to CO_2_. This can also be justified by the nearly two times higher experimental mass transfer coefficients of CH_4_ than CO_2_ recorded for this work. The reason for the gas flowrate favoring CO_2_ transport can be justified by the high driving force created at higher gas flow rates. The synthetic mixtures consisted of a large quantity of dissolved CO_2_. Thus, the degassed mixture had a large ratio of CO_2_, and a high gas flow rate was needed to create an enhanced driving force for the dissolubility of water-soluble CO_2_. The effect of the gas and liquid flow rates on the selectivity of the biogas components and the selective transport of CO_2_ and CH_4_ will be further discussed in terms of the mass transfer coefficients and transmembrane mass transport resistance in the following section.

### 3.4. Experimental and Theoretical Mass Transfer Analysis: Pseudo-Wetting Prediction

**Figure 7:**
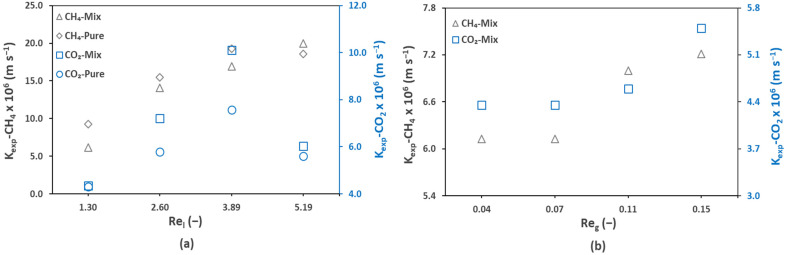
Experimental mass transfer coefficient of CH_4_ and CO_2_ in mix gas and pure gas effluents against (**a**) liquid Reynold, Re_l_ = 1.30–5.19, liquid flowrate, Q_l_ = 100–400 mL min^−1^ (**b**) gas Reynold, Re_g_ = 0.07–0.15, gas flowrate, Q_g_ = 50–200 mL min^−1^; T = 25 °C.

The overall experimental mass transfer coefficients (Equations (9)–(11)) of mix and pure effluents during the membrane degassing process were calculated and are presented here in Figure 7. In general, the CH_4_ mass transfer coefficients were higher than that of CO_2_. For example, at a Reynold number of 1.30 (Ql = 100 mL min^−1^), a value of 9.26 × 10^−6^ m s^−1^ was recorded for pure CH_4_ while for pure CO_2_, the value was 4.30 × 10^−6^ m s^−1^. In similar conditions, for mix CH_4_, K_exp_ of 6.13 × 10^−6^ m s^−1^ was recorded while for mix CO_2_, it was 4.35 × 10^−6^ m s^−1^. It can be observed from the above-mentioned values of K_exp_, for both pure and mix gas effluents, that K_exp_ of pure CH_4_ is nearly 1.5 times higher than that of mix CH_4_ while for CO_2_, it is nearly similar. As expected, the effect of the liquid and gas flow rates was dominant as can be seen from the figure. Both the liquid and gas flow rates favored the experimental mass transfer coefficient, except for CO_2_ at a liquid flow rate of 400 mL min^−1^ (Re_l_ = 5.19).

Table 6 presents a comparative analysis of the experimental mass transfer coefficient of CH_4_ from this work and the work from various other authors in various conditions. The porous membranes presented in this table had the same pore size of 0.04 µm, except for Sethunga et al., 2019 [32] (no pore size mentioned). This work recorded a coefficient of 1.93 × 10^−5^ m s^−1^ for CH_4_ against a liquid flow rate of 300 mL min^−1^ (V_l_ = 0.017 m s^−1^, Re_l_ = 3.89) and gas flow rate of 100 mL min^−1^ (V_g_ = 0.0020 m s^−1^/Re_g_=0.07). Jiménez-Robles et al., 2021 [37] (very low membrane area and gas velocity compared to this work), Cookney et al., 2016 [49] (lower liquid velocity compared to this work), McLeod et al., 2016 [51] (very high gas/liquid hydrodynamics), and Sethunga et al., 2019 [32] (very low membrane area compared to this work) reported lower experimental mass transfer coefficients when using sweep gas in PP membranes (such as for this work). Sanchis-Perucho et al., 2020 [36], reported a very low experimental mass transfer coefficient (0.38 × 10^−5^ m s^−1^) in comparison to our work, but they used a PDMS dense membrane (which induces a high membrane mass transfer resistance) with vacuum degassing. Henares et al., 2018 [43], recorded a value (1.94 × 10^−5^ m s^−1^) similar to our work using combined sweep gas and vacuum in a PP membrane at a relatively low liquid (0.013 m s^−1^) and high gas (0.0220 m s^−1^) velocity. Sethunga et al., 2019 [32], using sweep gas in a modified PVDF membrane, reported an experimental mass transfer coefficient of 2.50 × 10^−5^ m s^−1^, which is relatively high.

The overall theoretical mass transfer coefficient K_ov_ was also calculated (Equation (13)) using the local gas-side mass transfer coefficient (Equation (15)), local liquid-side mass transfer coefficient (Equation (16)), and mass transfer coefficient through the membrane (Equation (17)). It was confirmed (Table 7) that the main resistance to the mass transfer lies on the liquid side of the membrane, in case of the absence of pore wetting.

The overall theoretical mass transfer coefficient was compared with the overall experimental mass transfer coefficient K_exp_ (Table 7), which showed significant differences. For example, the overall theoretical mass transfer coefficient of CH_4_ was 1.49 × 10^−5^ m s^−1^, while the experimental one was 9.26 × 10^−6^ m s^−1^. In order to explain these differences, the hypothesis of a partial wetting of the membrane pores was supposed. The overall theoretical mass transfer coefficient was thus considered to be the ideal one (with no wetting) while the overall experimental mass transfer coefficient was the effective one (with pseudo-wetting). The effective membrane diffusivity (D_m,eff_) and wetted membrane mass transfer coefficient (K_m,w_) was then estimated by back substitution (Equations (18, 19) using the actual experimental mass transfer coefficient (K_exp_) and the results are presented in Table 7.

In Table 7, when the gas to liquid ratio is 1, the CH_4_ effective membrane diffusivity (4.75 × 10^−10^ m^2^ s^−1^) is nearly three times lower than that of the ideal membrane diffusivity (1.33 × 10^−7^ m^2^ s^−1^). For CO_2_, the effective membrane diffusivity (2.79 × 10^−9^ m^2^ s^−1^) was recorded as nearly two times lower than the ideal one (2.20 × 10^−7^ m^2^ s^−1^). The significant difference between the ideal and effective membrane diffusivity provides a clue about the possible pseudo-wetting of the membrane pores. Similarly, the pseudo-wetted membrane mass transfer coefficient (7.15 × 10^−7^ m s^−1^) of CH_4_ was nearly three times lower that of the non-wetted membrane mass transfer coefficient (2.08 × 10^−4^ m s^−1^). For CO_2_, the pseudo-wetted membrane mass transfer coefficient (4.36 × 10^−6^ m s^−1^) was nearly two times lower that of the non-wetted membrane mass transfer coefficient (3.44 × 10^−4^ m s^−1^).

**Figure 8:**
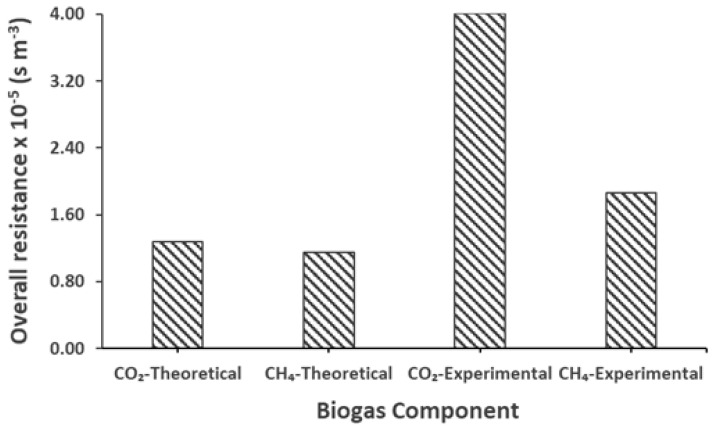
Pure CH_4_ and CO_2_ theoretical and experimental overall mass transfer resistance, Q_l_ = 100 mL min^−1^, Re_l_ = 1.30, Q_g_ = 100 mL min^−1^, Re_g_ = 0.07, T = 25 °C, P = 1 bar.

Membrane wetting can significantly increase the mass transfer resistance inside the membrane, causing a significant drop in the mass transfer coefficient [52,53,54]. The overall and experimental resistance was calculated using Equations (13) and (14), respectively, and are presented in Table 7. In light of the above-mentioned explanation of the existence of possible pseudo-wetting, Figure 8 presents the overall theoretical and experimental mass transfer resistance (s m^−3^) of the biogas during the degassing in the membrane contactor. It is obvious that the theoretical resistance was far lower than the experimental one. A comparison between the theoretical and experimental resistance clarifies that the actual and real resistance to the mass transfer was very high compared to the one measured theoretically, considering the no-wetting conditions. Yet again, here, we can justify the existence of pseudo-wetting. Additionally, we can observe that there is greater resistance to the mass transport of CO_2_ than CH_4_. Considering the ideal theoretical conditions, the difference between the resistance to CH_4_ and CO_2_ was small; however, a significant difference between the experimental resistance of CH_4_ and CO_2_ can be observed. This also explains the preference of CH_4_ over CO_2_ in the selective transmembrane transport. Furthermore, this effect could be observed for real AnMBR permeate in the following section.

### 3.5. Real AnMBR Permeate Dissolved Biogas Degassing 

This section presents the analysis and observations from using real AnMBR permeates in the membrane contactor degassing setup for biogas recovery. The AnMBR permeate initially consisted of 10–12 mg L^−1^ of dissolved CH_4_ and 40–60 mg L^−1^ of dissolved CO_2_ (Table 2). The amount of dissolved CH_4_ is in agreement with the amount reported by Sanchis-Perucho et al., 2020 [36] (12.01 mg L^−1^) for an AnMBR proto-type plant. As shown in Figure 2, the dissolved CH_4_ amount in the AnMBR permeate is very close to the amount measured theoretically for water as a solvent (13.7 mg L^−1^), and also to the amount used for the synthetic effluents (12.9 mg L^−1^) that were prepared for this work. Although various authors, such as Galib et al., 2016 [55], Smith et al., 2013 [56], Wu et al., 2017 [57], and Yeo et al., 2015 [58], reported dissolved CH_4_ super saturation degrees of 3 (54 mg L^−1^), 1.5, 1.2 (24.7 mg L^−1^), and 2.5 (35 mg L^−1^), respectively, for AnMBR permeate, it can be observed that the permeate from the AnMBR of this study seems to not be supersaturated. The absence of supersaturation could be justified by the amount of dissolved CH_4_ of the permeate used in this study (12 mg L^−1^), which is very close to the theoretical saturation value (13.7 mg L^−1^). The permeate at the exit of the anaerobic membrane recorded average values of 2.26 and 18.13 mg L^−1^ for TOC and COD, respectively. The TS and VS contents of the permeate were as low as 1.7 and 1.3 mg L^−1^, respectively. The particulate size in the permeate was lower than 0.04 μm (Microdyn Nadir^®^ ultrafiltration membrane pore size).

**Figure 9:**
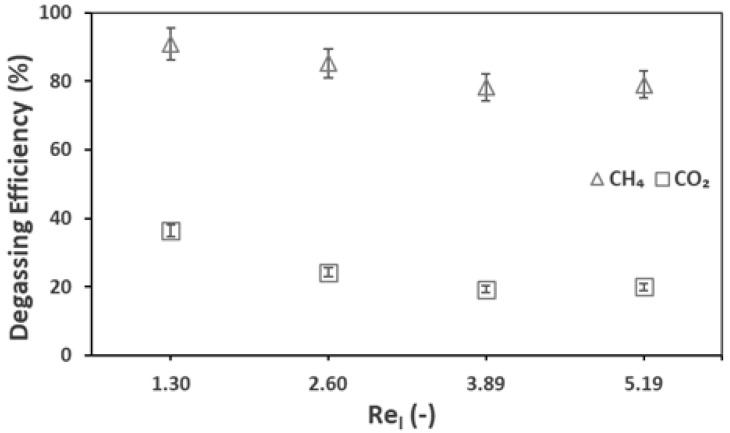
Membrane degassing efficiency for real lab-scale AnMBR permeates at liquid Reynold, Re_l_ = 1.30–5.19, liquid flow rate, Q_l_= 100–400 mL min^−1^ and gas Reynold, Re_g_ = 0.07, gas flow rate, Q_g_ = 100 mL min^−1^, T = 25 °C.

The degassing of the real effluent was performed at 25 °C, which is the original effluent temperature of the AnMBR. Figure 9 presents the membrane degassing efficiency for the biogas (CH_4_ and CO_2_). At a liquid flow rate of 100 mL min^−1^, (Re_l_ = 1.30), the dissolved methane content of the effluent at the membrane contactor outlet was as low as 0.98 mg L^−1^ while the degassing efficiency was very high (91%). The efficiency recorded for the synthetic mixed effluent was lower (89%) at this flow rate. A 13% efficiency drop was observed when the liquid flow rate was changed from 100 mL min^−1^ to 300 mL min^−1^ while in the case of synthetic mixed effluents, the efficiency drop was very low (5%) from the same flow rates. CO_2_ showed a comparatively low efficiency of 36.2% (39.3 mg L^−1^ of dissolved CO_2_ at the membrane contactor outlet) at a liquid flow rate of 100 mL min^−1^ while the recorded efficiency drop was 17% when the liquid flow rate was changed from 100 mL min^−1^ (Re_l_ = 1.30) to 300 mL min^−1^ (Re_l_ = 3.89). Referring to Section 3.2, the degassing efficiency was comparatively high for synthetic mixed effluents (62%) while the efficiency drop was nearly the same as that of the AnMBR permeate.

As mentioned earlier, the degassing process was monitored on both the gas and liquid sides. During the degassing process, the concentration of the desorbed biogas to the shell side of the membrane contactor was quantified using an X-stream gas analyzer. When the gas to liquid flow rate ratio was fixed at 1, initial CH_4_ and CO_2_ concentrations of 10.1 and 26.4 mg L^−1^, respectively, were recorded in the stripping gas stream.

**Figure 10:**
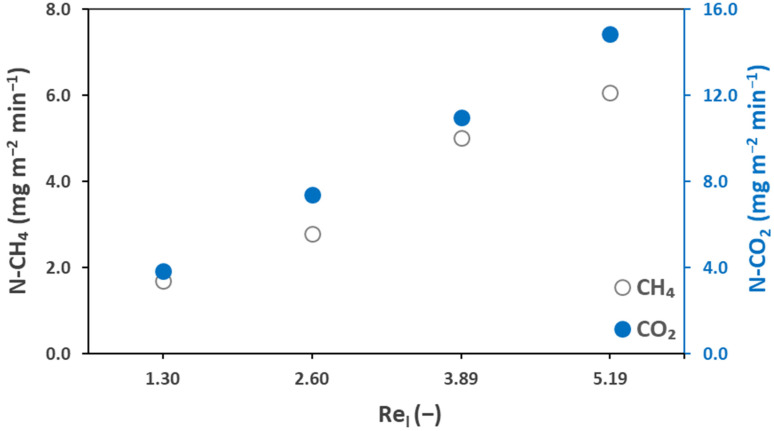
Biogas transmembrane flux for AnMBR permeates at liquid Reynold, Re_l_ = 1.30–5.19, liquid flow rate, Q_l_ = 100–400 mL min^−1^ and gas Reynold, Re_g_ = 0.07, gas flow rate, Q_g_ = 100 mL min^−1^, T = 25 °C.

Figure 10 presents the transmembrane flux of the biogas at different Reynold numbers. At a Reynold number of 5.19, flux values of 6.06 and 14.85 mg m^−2^ min^−1^ were recorded for CH_4_ and CO_2_, respectively. In similar conditions, the flux values recorded for the synthetic mixed effluents were 5.27 and 85.71 mg m^−2^ min^−1^ for CH_4_ and CO_2_, respectively. It is obvious from the data mentioned here that similar to the degassing efficiency, the CH_4_ flux was higher for the AnMBR permeate than for the synthetic mixed effluent (as discussed in Section 3.3). For CO_2_, the opposite effect was observed as the flux of the synthetic mixed effluent was very high compared to the flux from the AnMBR permeate. This seems to be due to the low dissolved CO_2_ contents of the AnMBR permeate in comparison to the synthetic effluent, which was prepared with a high CO_2_ content and in unfavorable conditions for CH_4_ degassing, which leads to a reduced driving force. The effect of the Reynold number on the transmembrane flux is very clear from the figure, as changing it from 1.29 to 5.19 increased the CH_4_ flux by 4.37 mg m^−2^ min^−1^. Similarly, for CO_2_, changing the Reynold from 1.29 to 5.19 increased the flux by 11 mg m^−2^ min^−1^, confirming the results established with the synthetic effluent.

The selective transport of CH_4_ over CO_2_ (CH_4_ flux/CO_2_ flux) in the real AnMBR permeate conditions was also analyzed. Detailed analysis of the selective transport of the synthetic effluents is presented in Section 3.3. For the real AnMBR permeate, a value of 1.20 was recorded for the selective transport of CH_4_ over CO_2_ at a gas to liquid flow rate ratio of 1. This value is very high compared to the one recorded for the mix (0.084) and pure (0.10) synthetic effluents in similar operating conditions. The higher value in real effluent conditions again corresponds to the similar amount of dissolved CH_4_ in both the synthetic and real effluents and the lower amount of dissolved CO_2_ in the real effluents.

**Figure 11:**
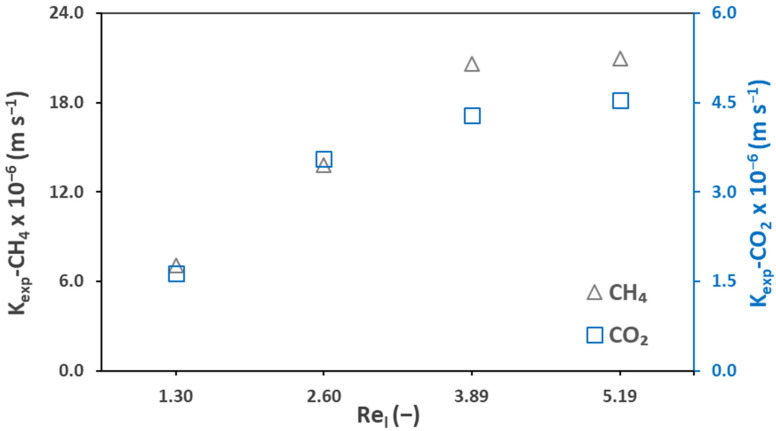
Experimental mass transfer coefficient of biogas for AnMBR permeates at liquid Reynold, Re_l_ = 1.30–5.19, liquid flow rate, Q_l_ = 100–400 mL min^−1^ and gas Reynold, Re_g_ = 0.07, gas flow rate, Q_g_ = 100 mL min^−1^, T = 25 °C.

Figure 11 represents the experimental mass transfer coefficients of the biogas against the liquid flow rate. At a liquid flow rate of 400 mL min^−1^ (Re_l_ = 5.19), experimental mass transfer coefficients of 2.1 × 10^−5^ m s^−1^ and 4.5 × 10^−6^ m s^−1^ were recorded for CH_4_ and CO_2_, respectively. A similar value of the CH_4_ experimental mass transfer coefficient (2.0 × 10^−5^ m s^−1^) was recorded for synthetic mixed effluent (as discussed in Section 3.4) in similar conditions. The reason might be the similar dissolved CH_4_ concentrations of the synthetic mixture and mixed effluents. For CO_2_, in similar conditions, the value of the experimental mass transfer coefficient was a higher (6.03 × 10^−6^ m s^−1^) for the synthetic mixed effluents; however, a higher value of 1.9 × 10^−05^ m s^−1^ was recorded for CO_2_ at a liquid flow rate of 300 mL min^−1^. The effect of the liquid flow rate is shown in Figure 11. The liquid flow rate favors the experimental mass transfer coefficient for both CH_4_ and CO_2_. By increasing the liquid flow rate from 100 mL min^−1^ (Re_l_ = 1.30) to 400 mL min^−1^ (Re_l_ = 5.19), an increase of 1.4 × 10^−5^ m s^−1^ in the CH_4_ experimental mass transfer coefficient was recorded.

**Figure 12:**
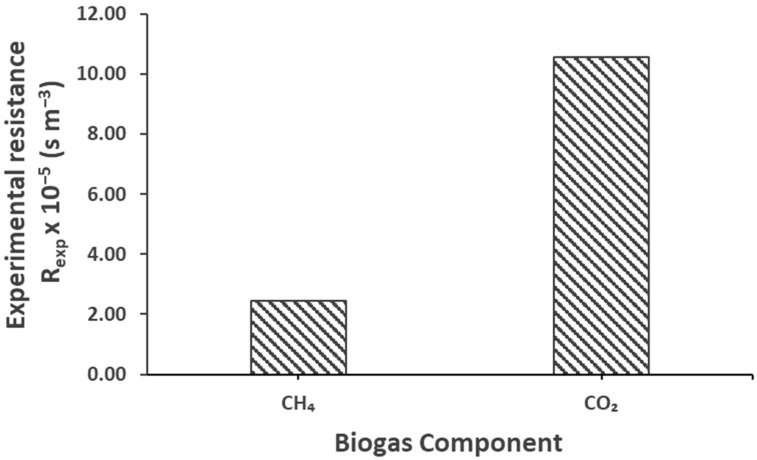
Experimental overall mass transfer resistance of biogas for real effluents at liquid Reynold, Re_l_ = 1.30, liquid flow rate, Q_l_ = 100 mL min^−1^ and gas Reynold, Re_g_ = 0.07, gas flow rate, Q_g_ = 100 mL min^−1^, T = 25 °C.

The experimental mass transfer resistance to the transport of biogas in the membrane contactor is presented here in Figure 12. The results revealed that the resistance to the mass transfer of CH_4_ from the real AnMBR permeate was very low compared to the resistance recorded for CO_2_. This high resistance to the transport of CO_2_ could be justified by its high solubility in the effluent (as the earlier findings of this work suggested the main resistance at the liquid side), which decreased its degassing potential compared to CH_4_. It should be noted that the low resistance and high transport of CH_4_ is favorable and desirable.

## 4. Conclusions

A membrane degassing system based on the porous hollow-fiber membrane contactor was developed and coupled directly with AnMBR to treat real AnMBR ultrafiltration permeate for the recovery of biogas. This study implemented a detailed approach to the analysis of the simultaneous recovery of both CH_4_ and CO_2_, first from synthetically prepared effluents and then from real AnMBR permeate. This work conducted an in-depth analysis of the biogas recovery from AnMBR permeates and presented promising and effective results compared to that reported before.

During the two hours of degassing operation, the membrane degassing setup was able to recover (out of the total dissolved amount) up to 93% CH_4_ and 83% CO_2_ at recovery rates of 0.12 and 4.41 mg L^−1^ min^−1^, respectively. The membrane contactor operated at a CH_4_ degassing efficiency of 95.7%, 89%, and 91% for pure gas effluent, mixed gas effluent, and real AnMBR permeate, respectively. The overall performance based on the obtained results was higher than the one reported before in other works considering nearly similar conditions. An interesting approach was implemented to observe the effect of the gas and liquid hydrodynamics on the selective transport of CH_4_ and CO_2_. This study revealed that an increase in the liquid flow rate favors CH_4_ transport over CO_2_ while an increase in the gas flow rate favors the transport of CO_2_. CH_4_ was observed to have a higher mass transfer coefficient than CO_2_, both theoretically and experimentally (considering the synthetic effluents and the real AnMBR permeate). This phenomenon was also validated by the higher transmembrane mass transfer resistance of CO_2_ than CH_4_ in all experimental and theoretical evaluations. The results also provided some evidence that partial pseudo-wetting of the membrane may occur.

Additional work is required to improve the membrane contactor’s performance for biogas recovery and to study the long-term effects. Further work investigating long-term operation of biogas degassing from real AnMBR permeate in porous and dense membranes with a particular focus on partial wetting and detailed fouling analysis is underway.

## Figures and Tables

**Table 1:** Characteristics of the real AnMBR permeate used in this study.

Parameter	Value
Temperature, T (°C)	25
pH (-)	7.0 ± 0.3
Total organic carbon, TOC (mg L^−1^)	2.2 ± 0.5
Chemical oxygen demand, COD (mg L^−1^)	18 ± 4
Total solids, TS (mg L^−1^)	1.7 ± 0.2
Volatile solids, VS (mg L^−1^)	1.3 ± 0.2
Dissolved methane, dCH_4_ (mg L^−1^)	11 ± 1
Dissolved carbon dioxide, dCO_2_ (mg L^−1^)	50 ± 10

**Table 2:** Membrane module specifications and operating conditions.

Parameter	Value
**Membrane Contactor**	
Membrane material	Polypropylene
Fiber inner diameter, d_i_ (m)	2.20 × 10^−4^
Fiber outer diameter, d_o_ (m)	3.00 × 10^−4^
Membrane thickness, δ (m)	0.4 × 10^−4^
Effective length of the fiber, L (m)	0.1132
Number of fibers, N	7400
Membrane pore diameter, d_p_ (m)	4.00 × 10^−8^
Effective inner membrane area, A_i_ (m^2^)	0.58
Effective outer membrane area, A_o_ (m^2^)	0.79
Lumen side volume, V_l_ (mL)	53
Shell side volume, V_g_ (mL)	78
Porosity, ε	40
Packing factor, ϕ	0.36
Tortuosity, τ ^a^	6.4
**Operating conditions**	
Operating configuration	Countercurrent
Operating temperature T (°C)	25
Liquid flowrate, Q_l_ (mL min^−1^)	100–400
Liquid velocity, V_l_ (m s^−1^)	5.93 × 10^−3^–2.37 × 10^−2^
Liquid Reynold number, Re_l_ (-)	1.29–5.19
Gas flowrate, Q_g_ (mL min^−1^)	50–200
Gas velocity, V_g_ (m s^−1^)	1.11 × 10^−3^–4.43 × 10 ^−3^
Gas Reynold number, Re_g_ (-)	3.70 × 10^−2^–1.48 × 10^−1^

^a^ τ $=\frac{{\left(2-\varepsilon \right)}^{2}}{\varepsilon }$ [41].

**Table 3:** Comparison of the membrane degassing efficiency of CH_4_ from various authors and from the present study at different liquid flowrates.

Reference	Effluent	Membrane	Operating Mode	V_l_ (m s^−1^)	Q_l_ (mL min^−1^)	V_g_ (m s^−1^)	Q_g_ (mL min^−1^)	Degassing Efficiency (%)
This Work	Saturated water	PP	Sweep gas	0.006	100	0.0020	100	95.7
This Work	Saturated water	PP	Sweep gas	0.024	400	0.0020	100	78.1
This Work	Saturated water	PP	Sweep gas	0.006	100	0.004	200	78.1
[36]	AnMBR prototype-plant	PDMS	Vacuum	-	833	-	-	45.0
[36]	AnMBR prototype-plant	PDMS	Vacuum	-	3333	-	-	18.0
[37]	Saturated water	PP	Sweep gas + vacuum	0.016	83	0.0004	8.3	84
[37]	Saturated water	PP	Sweep gas + vacuum	0.089	466	0.0004	8.3	42
[43]	EGSB	PP	Sweep gas + vacuum	0.013	68	0.0220	433	82
[43]	EGSB	PP	Sweep gas + vacuum	0.086	453	0.0220	433	45

**Table 4:** CH_4_ flux at different gas and liquid flow rates.

Q_l_ (mL min^−1^)/V_l_ (m s^−1^)/Re_l_	100/0.006/1.30	400/0.024/5.19
**Q_g_ (mL min^−1^)/V_g_ (m s^−1^)/Re_g_**	**Mix CH_4_ Flux (mg m^−2^ min^−1^)**	
50/0.001/0.04	1.47	-
100/0.002/0.07	1.71	5.27
200/0.004/0.15	1.93	-
	**Pure CH_4_ Flux (mg m^−2^ min^−1^)**	
100/0.002/0.07	3.77	11.07

**Table 5:** Comparison of CH_4_ transmembrane flux from various authors.

Ref.	Effluent	Membrane	Operating Mode	V_l_ (m s^−1^)	Q_l_ (mL min^−1^)	V_g_ (m s^−1^)	Q_g_ (mL min^−1^)	N (mg m^−2^ min^−1^)
This Work	Saturated water	PP	Sweep gas	0.024	400	0.0020	100	11.07
[36]	AnMBR prototype-plant	PDMS	Vacuum	-	833	-	-	2.41
[32]	Saturated water	PP	Sweep gas	0.1	-	-	20	9.63
[32]	Saturated water	Modified PVDF	Sweep gas	0.1	-	-	20	18.28

**Table 6:** Comparison of the CH_4_ experimental mass transfer coefficient (K_exp_), from various authors.

Ref.	Effluent	Membrane	Operating Mode	V_l_ (m s^−1^)	Q_l_ (mL min^−1^)	V_g_ (m s^−1^)	Q_g_ (mL min^−1^)	K_exp_ × 10 ^5^ (m s^−1^)
This Work	Saturated water	PP	Sweep gas	0.017	300	0.0020	100	1.93
[37]	Saturated water	PP	Sweep gas + vacuum	0.016	83	0.0004	8.3	1.65
[36]	AnMBR prototype-plant	PDMS	Vacuum	-	833	-	433-	0.38
[43]	EGSB	PP	Sweep gas + vacuum	0.013	68	0.0220		1.94
[49]	Saturated water	PP	Sweep gas	0.012	203	0.045	-	1.56
[51]	Saturated water	PP	Sweep gas	0.118	2000	0.018	1000	1.2
[32]	Saturated water	PP	Sweep gas	0.1	-	-	20	1.5
[32]	Saturated water	Modified PVDF	Sweep gas	0.1	-	-	20	2.5

**Table 7:** Prediction of the mass transport characteristics of biogas for wetted and pseudo-wetted membrane at P=1.01 bar, T= 25 °C.

Property	Q_g_/Q_l_	D_m,g_ (m^2^ s^−1^)	D_m,eff_ (m^2^ s^−1^)	K_g_ (m s^−1^)	K_l_ (m s^−1^)	K_m,nw_ (m s^−1^)	K_m,w_ (m s^−1^)	K_ov_ (m s^−1^)	K_exp_ (m s^−1^)
CH_4_	1	1.33 × 10^−7^	4.75 × 10^−10^	1.10 × 10^−4^	1.50 × 10^−5^	2.08 × 10^−4^	7.15 × 10^−7^	1.49 × 10^−5^	9.26 × 10^−6^
CO_2_	1	2.20 × 10^−7^	2.79 × 10^−9^	8.40 × 10^−5^	1.54 × 10^−5^	3.44 × 10^−4^	4.36 × 10^−6^	1.35 × 10^−5^	4.31 × 10^−6^

## Data Availability

Not applicable.

## Appendix A. Parameters for Local Mass Transfer Calculations

**Table A1:** Parameters used for local mass transfer calculations.

Parameter	Specie	Equation	Ref.
Gas diffusivity (m^2^ s^−1^) (in gas phase)	CO_2_,CH_4_,N_2_	$D_{g}=$$\frac{0.001858T^{3/2}{\left(\frac{1}{M_{1}}+\frac{1}{M_{2}}\right)}^{1/2}}{P\sigma_{A1,2}^{2}{Ω}_{D}}$ (1 ,2 = Gas components)	[59]
	Mix-effective	$D_{g}=\frac{1}{y_{2}^{\prime }/D_{1,2}+y_{3}^{\prime }/D_{1,3}}$$,\;y_{n}^{´}=\frac{y_{n}}{1-y_{1}}$, (1, 2, 3 … n = gas species)	
Gas diffusivity (m^2^ s^−1^) (in liquid phase)	CO_2_, CH_4_	$D_{l}=7.4\;10^{-8}\left(\frac{T{\left(\varphi_{2}M_{2}\right)}^{1/2}}{\mu_{2}V_{1}^{0.6}}\right)$ (1 = Gas solute, 2 = Liquid solvent)	
Gas diffusivity (m^2^ s^−1^) (porous membrane)	CO_2_, CH_4_	$\frac{1}{D_{m,g}}=\frac{1}{D_{g}}+\frac{1}{D_{kn}}$ $D_{kn}=\frac{1}{3}d_{p}\sqrt{\frac{8RT}{\pi M}}$	[45]
Density (Kg m^−3^)	Gas mixture	$\rho_{g,\;mix}=\frac{\left(y_{CH_{4}}M_{CH_{4}}+y_{CO_{2}}M_{CO_{2}}+y_{N_{2}}M_{N_{2}}\right)\;P_{g}}{0.082\;T_{g}}$	Ideal Gas Law
	Water	$\rho_{l}=10^{3}\;\left(0.863559+1.21494\;10^{-3}\;T_{L}-2.5708\;10^{-3}\;T_{L}\right)$	[60]
Viscosity (Kg m^−1^ s^−1^)	Gas mixture	$\mu_{g,mix}=\overset{3}{\underset{i=1}{\sum }}\frac{y_{i}\mu_{i}}{\sum_{j=1}^{3}y_{i}\psi_{ij}}$ $,\;\psi_{ij}={\left(\frac{M_{j}}{M_{i}}\right)}^{0.5}$ $,\;\psi_{ji}=\frac{1}{\psi_{ij}}$	[61]
	Water	$\mu_{l}=10^{-6}\;\rho_{l}\exp\left(-3.28285\frac{456.029}{T_{l}-154.576}\right)$	[60]
